# Churchill: an ultra-fast, deterministic, highly scalable and balanced parallelization strategy for the discovery of human genetic variation in clinical and population-scale genomics

**DOI:** 10.1186/s13059-014-0577-x

**Published:** 2015-01-20

**Authors:** Benjamin J Kelly, James R Fitch, Yangqiu Hu, Donald J Corsmeier, Huachun Zhong, Amy N Wetzel, Russell D Nordquist, David L Newsom, Peter White

**Affiliations:** Center for Microbial Pathogenesis, The Research Institute at Nationwide Children’s Hospital, 700 Children’s Drive, Columbus, OH 43205 USA; Department of Pediatrics, College of Medicine, The Ohio State University, Columbus, Ohio USA

## Abstract

While advances in genome sequencing technology make population-scale genomics a possibility, current approaches for analysis of these data rely upon parallelization strategies that have limited scalability, complex implementation and lack reproducibility. Churchill, a balanced regional parallelization strategy, overcomes these challenges, fully automating the multiple steps required to go from raw sequencing reads to variant discovery. Through implementation of novel deterministic parallelization techniques, Churchill allows computationally efficient analysis of a high-depth whole genome sample in less than two hours. The method is highly scalable, enabling full analysis of the 1000 Genomes raw sequence dataset in a week using cloud resources. http://churchill.nchri.org/.

**Electronic supplementary material:**

The online version of this article (doi:10.1186/s13059-014-0577-x) contains supplementary material, which is available to authorized users.

## Background

Next generation sequencing (NGS) has revolutionized genetic research, enabling dramatic increases in the discovery of new functional variants in syndromic and common diseases [1]. NGS has been widely adopted by the research community [2] and is rapidly being implemented clinically, driven by recognition of its diagnostic utility and enhancements in quality and speed of data acquisition [3]. However, with the ever-increasing rate at which NGS data are generated, it has become critically important to optimize the data processing and analysis workflow in order to bridge the gap between big data and scientific discovery. In the case of deep whole human genome comparative sequencing (resequencing), the analytical process to go from sequencing instrument raw output to variant discovery requires multiple computational steps (Figure S1 in Additional file 1). This analysis process can take days to complete, and the resulting bioinformatics overhead represents a significant limitation as sequencing costs decline and the rate at which sequence data are generated continues to grow exponentially.

Current best practice for resequencing requires that a sample be sequenced to a depth of at least 30× coverage, approximately 1 billion short reads, giving a total of 100 gigabases of raw FASTQ output [4]. Primary analysis typically describes the process by which instrument-specific sequencing measures are converted into FASTQ files containing the short read sequence data and sequencing run quality control metrics are generated. Secondary analysis encompasses alignment of these sequence reads to the human reference genome and detection of differences between the patient sample and the reference. This process of variant detection and genotyping enables us to accurately use the sequence data to identify single nucleotide polymorphisms (SNPs) and small insertions and deletions (indels). The most commonly utilized secondary analysis approach incorporates five sequential steps: (1) initial read alignment; (2) removal of duplicate reads (deduplication); (3) local realignment around known indels; (4) recalibration of the base quality scores; and (5) variant discovery and genotyping [5]. The final output of this process, a variant call format (VCF) file, is then ready for tertiary analysis, where clinically relevant variants are identified.

Of the phases of human genome sequencing data analysis, secondary analysis is by far the most computationally intensive. This is due to the size of the files that must be manipulated, the complexity of determining optimal alignments for millions of reads to the human reference genome, and subsequently utilizing the alignment for variant calling and genotyping. Numerous software tools have been developed to perform the secondary analysis steps, each with differing strengths and weaknesses. Of the many aligners available [6], the Burrows-Wheeler transform based alignment algorithm (BWA) is most commonly utilized due to its accuracy, speed and ability to output Sequence Alignment/Map (SAM) format [7]. Picard and SAMtools are typically utilized for the post-alignment processing steps and produce SAM binary (BAM) format files [8]. Several statistical methods have been developed for variant calling and genotyping in NGS studies [9], with the Genome Analysis Toolkit (GATK) amongst the most popular [5].

The majority of NGS studies combine BWA, Picard, SAMtools and GATK to identify and genotype variants [1]. However, these tools were largely developed independently, contain a myriad of configuration options and lack integration, making it difficult for even an experienced bioinformatician to implement them appropriately. Furthermore, for a typical human genome, the sequential data analysis process (Figure S1 in Additional file 1) can take days to complete without the capability of distributing the workload across multiple compute nodes. With the release of new sequencing technology enabling population-scale genome sequencing of thousands of raw whole genome sequences monthly, current analysis approaches will simply be unable to keep up. These challenges create the need for a pipeline that simplifies and optimizes utilization of these bioinformatics tools and dramatically reduces the time taken to go from raw reads to variant calls.

## Results

Churchill fully automates the analytical process required to take raw sequence data through the complex and computationally intensive process of alignment, post-alignment processing and genotyping, ultimately producing a variant list ready for clinical interpretation and tertiary analysis (Figure S2 in Additional file 1). Each of these steps was optimized to significantly reduce analysis time, without downsampling and without making any sacrifices to data integrity or quality (see Materials and methods). At the heart of Churchill’s parallelization strategy is the development of a novel deterministic algorithm that enables division of the workflow across many genomic regions with fixed boundaries (subregions) (Figure S3 in Additional file 1). This division of work, if naively implemented, would have major drawbacks: read pairs spanning subregional boundaries would be permanently separated, leading to incomplete deduplication and variants on boundary edges would be lost. To overcome this challenge, Churchill utilizes both an artificial chromosome, where interchromosomal or boundary-spanning read pairs are processed, and overlapping subregional boundaries, which together maintain data integrity and enable significant performance improvements (Figures S4 and S5 in Additional file 1).

### Performance comparisons of parallelization strategies

**Figure 1:**
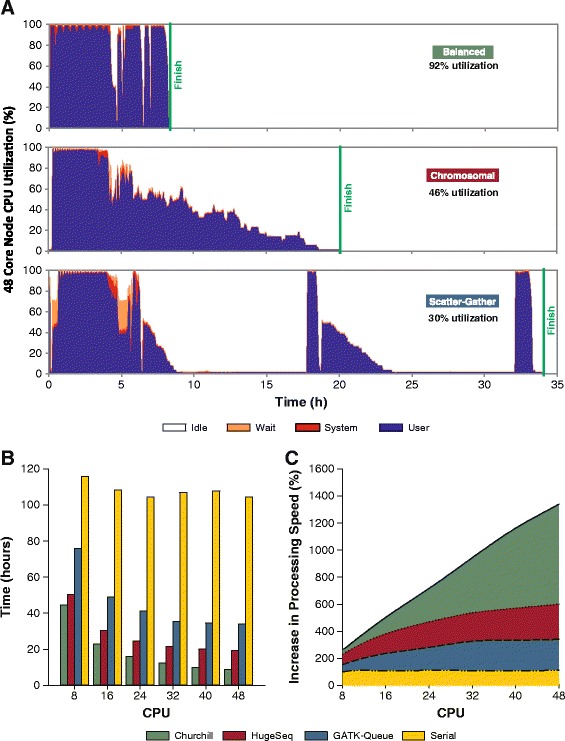
**Churchill optimizes load balancing, resulting in improved resource utilization and faster run times.** Three different strategies for parallelization of whole genome sequencing secondary data analysis were compared: balanced (utilized by Churchill), chromosomal (utilized by HugeSeq) and scatter-gather (utilized by GATK-Queue). The resource utilization, timing and scalability of the three pipelines were assessed using sequence data for a single human genome sequence dataset (30× coverage). **(A)** CPU utilization was monitored throughout the analysis process and demonstrated that Churchill improved resource utilization (92%) when compared with HugeSeq (46%) and GATK-Queue (30%). **(B)** Analysis timing metrics generated with 8 to 48 cores demonstrated that Churchill (green) is twice as fast as HugeSeq (red), four times faster than GATK-Queue (blue), and 10 times faster than a naïve serial implementation (yellow) with in-built multithreading enabled. **(C)** Churchill scales much better than the alternatives; the speed differential between Churchill and alternatives increases as more cores in a given compute node are used.

The parallelization approach adopted by Churchill overcomes the limitation of parallelization by chromosome, enabling a load balanced and independent execution of the local realignment, deduplication, recalibration and genotyping steps (Figure 1). The timing of each of these steps decreases in a near-linear manner as Churchill efficiently distributes the workload across increasing compute resources. Using a typical human genome data set, sequenced to a depth of 30×, the performance of Churchill’s balanced parallelization was compared with two alternative BWA/GATK based pipelines: GATK-Queue utilizing scatter-gather parallelization [5] and HugeSeq utilizing chromosomal parallelization [10]. The parallelization approach adopted by Churchill enabled highly efficient utilization of system resources (92%), while HugeSeq and GATK-Queue utilize 46% and 30%, respectively (Figure 1A). As a result, using a single 48-core server (Dell® R815), Churchill is twice as fast as HugeSeq, four times faster than GATK-Queue, and 10 times faster than a naïve serial implementation with in-built multithreading enabled (Figure 1B). Furthermore, Churchill scales highly efficiently across cores within a single server (Figure 1C).

**Figure 2:**
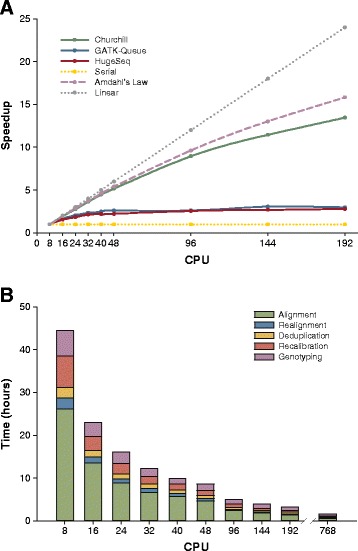
**Churchill scales efficiently, enabling complete secondary analysis to be achieved in less than two hours.** The capability of Churchill, GATK-Queue and HugeSeq to scale analysis beyond a single compute node was evaluated. **(A)** Fold speedup as a function of the number of cores used was assessed across a cluster of four Dell® R815 servers with Churchill (green), GATK-Queue (blue), HugeSeq (red) and serial analysis (yellow). For comparison, the linear speedup (grey) and that predicted by Amdahl’s law (purple) assuming a one-hour sequential time are also included [11]. Churchill’s scalability closely matches that predicted by Amdahl’s law, achieving in excess of a 13-fold speedup between 8 and 192 cores. In contrast, both HugeSeq and GATK-Queue showed modest improvements in speed between 8 and 24 cores (2-fold), with a maximal 3-fold speedup being achieved with 48 cores, and no additional increase in speed beyond 48 cores. **(B)** Timing results for different steps of the Churchill pipeline were assessed with increasing numbers of cores. Complete human genome analysis was achieved in three hours using an in-house cluster with 192 cores and in 100 minutes at the Ohio Supercomputer Center (Glenn Cluster utilizing 700 cores). Results were confirmed using both the Pittsburgh Supercomputing Center and Amazon Web Services EC2.

The capability of Churchill to scale beyond a single compute node was then evaluated (Figure 2). Figure 2A shows the scalability of each pipeline across a server cluster with fold speedup plotted as a function of the number of cores used. It is evident that Churchill’s scalability closely matches that predicted by Amdahl’s law [11], achieving a speedup in excess of 13-fold between 8 and 192 cores. In contrast, both HugeSeq and GATK-Queue showed modest improvements between 8 and 24 cores (2-fold), reaching a maximal 3-fold plateau at 48 cores. Churchill enabled resequencing analysis to be completed in three hours using an in-house cluster with 192 cores (Figure 2B). Simply performing alignment and genotyping (without deduplication, realignment, or recalibration) required twice the number of cores to achieve a similar analysis time using CrossBow [12]. Utilization of Churchill on both the Ohio Supercomputer Center’s Glenn Cluster (768 cores over 96 nodes) and on Amazon Web Services (AWS) Elastic Compute Cloud (EC2) (768 cores over 24 CR1 instances) enabled analysis completion in less than 1 hour 50 minutes.

**Figure 3:**
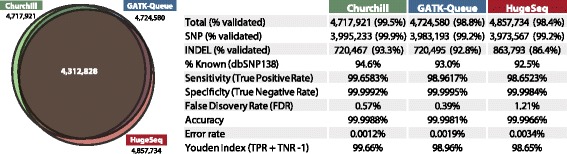
**The performance of Churchill does not come at the sacrifice of data quality.** The final VCF output of Churchill (green), GATK-Queue (blue) and HugeSeq (red) was compared and evaluated against the National Institute of Standards and Technology (NIST) benchmark SNP and indel genotype calls generated by the Genome in a Bottle Consortium (GIAB) [13]. The Venn diagram shows a high degree of concordance between the three pipelines. Churchill identified the highest number of validated variants from the approximately 2.9 million calls in the GIAB dataset, for both SNPs (99.9%) and indels (93.5%), and had the highest overall sensitivity (99.7%) and accuracy (99.9988%). The Youden index (or J statistic), a function of sensitivity (true positive rate) and specificity (true negative rate), is a commonly used measure of overall diagnostic effectiveness [14].

The output of Churchill was validated using the recently released National Institute of Standards and Technology (NIST) benchmark SNP and indel genotype calls generated by the Genome in a Bottle (GIAB) Consortium [13]. FASTQ files from the 1000 Genomes CEU female NA12878 were analyzed using Churchill, GATK-Queue and HugeSeq, all using the GATK UnifiedGenotyper algorithm for variant calling and genotyping, and resulting VCF files were compared (Figure 3). While there is a high degree of concordance between the three pipelines, Churchill produced the highest percentage of validated variant calls, for both SNPs (99.9%) and indels (93.3%), and had the highest overall sensitivity (99.7%) and accuracy (99.9988%). GATK-Queue had slightly higher specificity than Churchill, and the lowest false discovery rate (0.39%), but failed to identify approximately 20,000 validated variants found by Churchill. Of the three pipelines Churchill had the highest diagnostic effectiveness (99.66%), followed by GATK-Queue (98.96%) and HugeSeq (98.65%), as assessed by the Youden Index [14].

### Resource utilization and performance in the cloud

**Figure 4:**
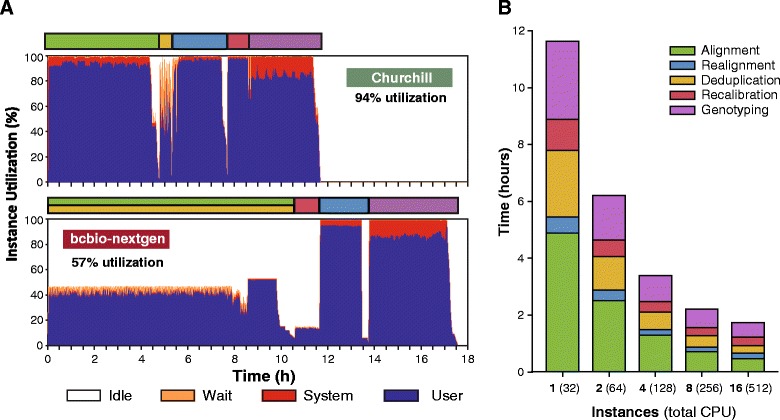
**Churchill enables rapid secondary analysis and variant calling with GATK HaplotypeCaller using cloud computing resources.** Analysis of raw sequence data for a single human genome sequence dataset (30× coverage) was compared using Churchill and bcbio-nextgen, with both pipelines utilizing BWA-MEM for alignment and GATK HaplotypeCaller for variant detection and genotyping. **(A)** CPU utilization on a single r3.8xlarge AWS EC2 instance (32 cores) was monitored throughout the analysis process and demonstrated that Churchill improved resource utilization (94%) when compared with bcbio-nextgen (57%), enabling the entire analysis to be completed in under 12 hours with a single instance. **(B)** Unlike bcbio-nextgen, Churchill enables all steps of the analysis process to be efficiently scaled across multiple compute nodes, resulting in significantly reduced run times. With 16 AWS EC2 instances the entire analysis could be completed in 104 minutes, with the variant calling and genotyping with GATK HaplotypeCaller stage taking only 24 minutes of the total run time.

The capability of Churchill to perform whole genome variant discovery and genotyping via local re-assembly of haplotypes was assessed using AWS cloud compute resources and the GATK HaplotypeCaller algorithm for variant discovery and genotyping [15]. For comparison purposes, the performance of Churchill on AWS was compared with bcbio-nextgen, a python toolkit that provides a distributed multi-architecture pipeline that automates variant calling [16]. Both pipelines were setup to utilize BWA-MEM [17] for alignment and GATK HaplotypeCaller for variant detection and genotyping to analyze raw sequence data for a human whole genome sequence dataset (30× coverage). CPU utilization on a single r3.8xlarge AWS EC2 instance (32 cores) was monitored throughout the analysis run. The results demonstrated that Churchill had significantly greater resource utilization (94%) than bcbio-nextgen (57%), enabling the entire analysis to be completed in under 12 hours with a single instance (Figure 4A). The initial phase of bcbio-nextgen execution uses a shell pipeline of BWA-MEM, samblaster [18], samtools and sambamba to perform alignment, mark duplicates, convert SAM to BAM, and sort the resulting BAM data. However, during this phase of processing, less than 50% CPU utilization was observed (Figure 4A).

Churchill enables all steps of the analysis process to be efficiently scaled across multiple AWS instances, resulting in significantly reduced run times (Figure 4B). With 16 AWS EC2 instances the entire analysis could be completed in 104 minutes, with the variant calling and genotyping with GATK HaplotypeCaller stage taking only 24 minutes. In contrast, using the default options of the bcbio-nextgen workflow, alignment and deduplication is parallelized by using the built-in multi-threading capabilities of BWA and sambamba, and as such it is limited in scalability to the number of cores available on a single machine. Next, the bcbio-nextgen software uses sambamba to index the single BAM resulting from the previous phase. Again this processing is limited to a single process that cannot scale beyond a single machine.

### Analysis of the 1000 Genomes Project on the cloud

**Figure 5:**
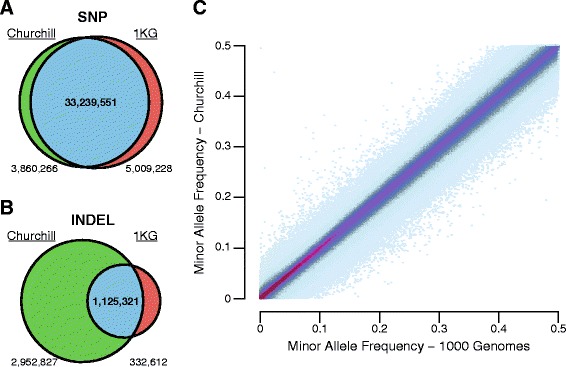
**Churchill enables population-scale whole human genome sequence analysis.** Churchill was used to analyze 1,088 of the low-coverage whole-genome samples that were included in ‘phase 1’ of the 1000 Genomes Project (1KG). Raw sequence data for the entire population were used to generate a single multi-sample VCF in 7 days using 400 AWS EC2 instances (cc2.8xlarge spot instances). The resulting Churchill filtered VCF (green) was then compared to the 1KG Consortium’s VCF (red), with Churchill calling 41.2 million variants and the 1KG VCF file containing 39.7 million. The two VCF file sets had a total of 34.4 million variant sites in common. **(A)** There were 33.2 million SNPs called in common, with validation rates against known SNPs being highly similar: 52.8% (Churchill) and 52.4% (1KG). **(B)** Churchill called three-fold more indels, of which 19.5% were known compared with 12.5% in the 1KG indel set. The indels unique to Churchill have a seven-fold higher rate of validation with known variants than those unique to 1KG. **(C)** Minor allele frequencies were compared for the 34.3 million variants with the same minor allele and a density binned scatter plot was produced (scaled from low (light blue) to high (purple) density frequencies). The results from Churchill and the original 1KG analysis demonstrated highly concordant minor allele frequencies (R^2^ = 0.9978, *P*-value <2.2e-16).

In order to demonstrate Churchill’s utility for population-scale genomic analysis, 1,088 low-coverage whole-genome samples from ‘phase 1’ of the 1000 Genomes Project (1KG) were analyzed, including calling variants with GATK’s UnifiedGenotyper on all samples simultaneously to generate a multi-sample final VCF. The entire analysis was completed in less than 7 days using 400 AWS EC2 instances (cc2.8xlarge spot instances) and the total analysis cost was approximately $12,000, inclusive of data storage and processing. Churchill identified 41.2 million variants versus 1KG’s 39.7 million (Figure 5). The two call sets had 34.4 million variant sites in common, of which 34.3 million had the same minor allele with highly similar frequencies (Pearson’s correlation coefficient of 0.9978, *P*-value <2.2e-16; Figure 5C). The results were validated against previously identified variants (dbSNP build 138, excluding those from the 1KG submission). SNP validation rates were similar, 52.8% (Churchill) and 52.4% (1KG). However, due to improvements in indel calling since the original 1KG analysis, Churchill called three-fold more indels with a higher rate of validation (19.5% versus 12.5%). Of the indels unique to Churchill, a seven-fold higher rate of validation was observed compared with those unique to 1KG. Of the GIAB consortium’s validated indel dataset [13], 81.5% were observed in the Churchill analysis in contrast to 43.9% with the 1KG analysis. Churchill called approximately 71% of the 99,895 novel validated indels in the GIAB NA12878 dataset (those not found in the 1KG analysis) with alternative allele frequencies as high as 100% (mean 40.2%).

## Discussion

The Churchill parallelization strategy optimizes utilization of available compute resources and scales in a near linear fashion, enabling population-scale genome analysis to be performed cost-effectively using cloud resources. Churchill brings together the most commonly utilized tools in a single pipeline using currently accepted best practices for discovery of genetic variation, fully automating alignment, deduplication, local realignment, base quality score recalibration, variant calling and genotyping. By carefully exploring interdependencies among different subtasks, Churchill achieves high levels of parallelism and completes reproducible data analysis in a fraction of the time, without sacrificing data quality or integrity.

### Churchill demonstrates deterministic analysis behavior

A parallel program is deterministic if, for a given input, every execution of the program produces identical externally visible output [19]. Therefore, for a parallel pipeline performing whole genome resequencing analysis, these criteria for determinism would be met if, given a set of raw sequence data as input, every execution of the program produces identical variant calls and genotypes as the final output. Not only are the results of Churchill analysis reproducible when executed with the same number of subregions, but Churchill analysis is deterministic; regardless of the scale of parallelization the final result is identical, making Churchill an optimal solution for clinical applications. Other parallelization strategies fail to achieve this level of reproducibility or make sacrifices in data quality for speed. Strikingly, non-determinism can be introduced at virtually every step in the analysis if configuration parameters are not carefully selected. For example, the developers of GATK recognize that results are non-deterministic when using built-in multithreading options and recommend disabling multithreading if absolute determinism is required at the expense of significantly increased run time. Moreover, GATK’s default use of downsampling can also result in differing output. Parallelism in Churchill does not utilize GATK multithreading, nor does it perform downsampling by default. Churchill provides the repeatability of results necessitated in clinical sequencing applications, and the deterministic behavior removes the potential for inconsistencies in repeat analysis or in larger studies where analysis is performed at multiple locations.

### Churchill eliminates interdependencies among analysis steps while maintaining a best-practice implementation

In order to efficiently distribute the analysis workflow, we developed a strategy to equally divide the genome into multiple subregions and to process each of those segments independently, creating an ‘embarrassingly parallel’ computation (Figures S2 and S4 in Additional file 1). Nearly all inter-process dependencies in the workflow have been removed, including elimination of two major merge points in the workflow: before deduplication and before assembly of the covariates table for base quality score recalibration.

Deduplication requires the entire set of reads in sorted order so that any number of read pairs that have identical mappings can be reduced to a single pair [5]. In parallelization of this deduplication process by subregions, mapping information of these read pairs must be kept together. Most read pair distances will be normally distributed around a given insert size that falls within the boundaries of a given subregion. Inherently there will be outliers that could represent sequencing artifacts or improper mappings, but in many cases read pairs with large insert sizes and those with mates mapped to different chromosomes provide important information about possible interchromosomal rearrangement (translocations). For example, the Catalogue Of Somatic Mutations In Cancer (COSMIC v70, August 2014) contains over 10,000 gene fusions known to be associated with benign and malignant tumors, many of which have been shown to play key roles in cancer initiation [20]. The clinical relevance of interchromosomal reads is further highlighted by the fact that gene fusions can be linked to clinical outcomes; for example, the presence of the BCR-ABL1 fusion is a powerful prognostic indicator in both pediatric [21] and adult leukemias [22]. As such, interchromosomal reads are properly handled during Churchill’s parallel processing. The addition of an artificial chromosome strictly for reads spanning subregions (including interchromosomal reads) allows for parallelized deduplication without the need for a costly merge step. In contrast, HugeSeq chromosomal parallelization breaks correspondence between read pairs that are not mapped to the same chromosome, preventing appropriate deduplication and reducing data quality. The authors of HugeSeq recognized this limitation in their approach, stating that parallelization of the interchromosomal read detection process was not possible [10]. One limitation of Churchill is that is does not currently automate the process of structural variant calling. While both the HugeSeq and bcbio-nextgen pipelines do, they do so with limited levels of parallelism (bcbio-nextgen is capable of parallelizing some steps in the structural variant calling process by chromosome). The Churchill parallelization algorithm does create an additional single BAM file containing all interchromosomal read pairs and those spanning subregions, allowing a user to concentrate computational resources on only those read pairs likely to identify a structural variant. Utilization of the subregion approach for the parallelization of structural variant calling is an active area of future development for the Churchill pipeline.

The second point at which different segments are codependent occurs during base quality score recalibration. Best practices suggest that a true measure of base qualities requires examination of covariates across the entire sample to provide empirically accurate base quality scores for each base in every read, and correct for multiple error covariates [15]. Churchill accomplishes this by generating covariate tables for each subregion and merging them into a single recalibration table for the entire sample. Recalibration is then applied in parallel to each subregion, producing identical results to recalibration applied to a single merged BAM of the entire genome. Furthermore, by avoiding downsampling at this stage, and taking into account qualities of every base for a given sample, identical results will be produced every time recalibration is performed. By contrast, HugeSeq applies the GATK count covariates function by chromosome, resulting in incomplete information about the quality score distribution, thereby reducing the effectiveness of the recalibration process.

### Churchill enables highly scalable parallelization and improves computational efficiency

In addition to faster performance, Churchill creates more independent processes and eliminates costly single-threaded merge steps, leading to optimized resource utilization and efficient load balancing (Figure 1A). Moreover, given the memory intensive nature of NGS analysis, the memory load can be efficiently spread amongst multiple machines. Churchill’s unique ability to analyze multiple chromosomal subregions in parallel enables it to efficiently scale with many hundreds of parallel processes, with scalability only limited by the need for a few synchronization points and the inherently serial steps (for example, deduplication cannot start until all FASTQ file pairs have been aligned), while alternative pipelines failed to scale efficiently beyond 24 parallel processes (Figure 2A). As a result of these improvements in scalability and efficiency, Churchill enables completion of an entire whole genome analysis, from raw sequence reads to a recalibrated VCF file, in less than two hours with either UnifiedGenotyper (Figure 2B) or HaplotypeCaller (Figure 4B).

Through utilization of alternative strategies for parallelization, GATK-Queue and HugeSeq achieve a moderate degree of parallelism and speedup [5,10]. GATK-Queue processes raw reads from multiple unaligned BAM files in parallel; realignment, base quality score recalibration, and genotyping are performed on multiple sub-chromosomal ‘intervals’ to achieve a high degree of parallelism. However, deduplication is carried out on a single merged BAM file and its workflow requires merging of all BAM files after realignment and after recalibration. These three lengthy single-threaded processes counteract the savings achieved through the sub-chromosomal interval parallelism, and average CPU utilization is less than 30% throughout the run (Figure 1A). The HugeSeq pipeline performs faster than GATK-Queue by performing parallelization at the chromosome level, thereby circumventing the BAM merging processes. However, this approach results in suboptimal results due to inappropriate deduplication of interchromosomal reads and a failure to consider all base qualities simultaneously during recalibration. Additionally, parallelization by chromosome limits scalability and suffers from poor load balancing due to the fact that human chromosomes vary greatly in size (Figure S3 in Additional file 1).

Improved performance was observed with the bcbio-nextgen pipeline, but elements of the parallelization strategy implemented by this software have similar limitations as GATKQ. The alignment, deduplication and BAM indexing steps are parallelized by using the built-in multi-threading capabilities of BWA and sambamba, producing a single merged BAM file, and as such limits parallelization of these steps to a single machine. This merge requirement of the bcbio-nextgen pipeline is avoided by Churchill via independent processing of reads spanning subregions in an artificial chromosome. The streaming deduplication approach utilized by sambamba does avoid Picard Tools’ deduplication requirement to read alignment results from disk and may result in a modest improvement in Churchill’s performance by reducing input/output (I/O). However, Churchill’s highly efficient parallelized deduplication strategy enables that stage of the analysis process to be completed in as little as 10 minutes. bcbio-nextgen parallelizes variant calling by partitioning the genome into regions that can be processed simultaneously. These regions are bounded by spans of the genome that contain no callable reads in any of the samples that are being processed. Although this approach is superior to parallelizing by chromosome and enables parallelization across multiple machines, it is still subject to processing regions of differing sizes, which performs and scales less well than Churchill, which utilizes regions of equal size, thereby achieving optimal load balancing and highly efficient resource utilization (Figure 4A).

The Churchill pipeline is currently reliant upon a shared file-system for storage of the input data, intermediate files produced during processing, and the final output files. This file-system is a possible performance bottleneck during processing depending on the infrastructure supporting it and the amount of other computational resources available. Streaming shell pipelines, distributed file-systems, and distributed processing frameworks offer the opportunity to further enhance Churchill’s efficiency, and are a priority for future work. These approaches to reducing disk I/O and network traffic will greatly benefit from a parallelization strategy like that offered by Churchill, since they would otherwise be limited to the computational resources available on a single computer. Churchill effectively distributes the CPU burden of NGS data processing by leveraging the CPU/memory resources available on individual computers within a cluster, and in a similar way could also allow for the distribution of I/O by leveraging the local I/O resources available on individual computers.

### Balanced parallelization with Churchill dramatically speeds up whole genome variant discovery and genotyping via local re-assembly of haplotypes

Haplotype-based variant detection methods, such as FreeBayes [23] and HaplotypeCaller [15], in which variant discovery and genotyping are performed by local re-assembly of haplotypes, may reduce false positive calls due to errors in short read alignment, but are considerably more computationally expensive than methods which operate on a single position at a time. In collaboration with Intel®, the Broad Institute recently developed a set of hardware-based optimizations for the PairHMM algorithm in HaplotypeCaller enabling them to reduce the time to analyze a single genome from three days to one day (a three-fold speedup). Utilization of Churchill’s balanced parallelization approach, in combination with AWS EC2 instances equipped with Intel Xeon® processors that can utilize the HaplotypeCaller routines optimized for Intel® Advanced Vector Extensions, enabled whole genome variant calling and genotyping in 24 minutes (a 60-fold speedup; Figure 4B). This resulted in a similar run time performance as UnifiedGenotyper (Figure 2) and enabled complete genome analysis in 1 hour 44 minutes using on-demand Cloud resources, without any sacrifice in data quality (Figure 4). While HaplotypeCaller is a more sophisticated algorithm than UnifiedGenotyper, it has been reported that the HaplotypeCaller indels have an increased false discovery rate [24] and significantly lower validation rates for both SNP and indel calls than UnifiedGenotyper [25]. As such, Churchill currently provides users with options for variant discovery and genotyping with UnifiedGenotype, HaplotypeCaller or FreeBayes.

### Churchill enables rapid clinical genomic analysis

Routine adoption of NGS clinically has been impeded by the complexity of the bioinformatics and the lack of a data analysis solution that is simple, fast and accurate [26]. Churchill eliminates the genomic analysis bottleneck for a clinical laboratory, transforming a complex workflow to a single command while observing currently accepted best practices for discovery of genetic variation. The entire secondary analysis workflow (from FASTQ to VCF) for a single sample can be completed in less than an hour for an exome or targeted panel and under 2 hours for a whole genome. The speed at which Churchill is able to complete NGS analysis will have a major impact in the clinic where fast turnaround can be essential for diagnosis of genetic disease. For instance, rapid diagnosis is critical for newborns with suspected monogenic diseases, where diagnosis is confounded by ambiguous symptoms and progression is rapid, frequently leading to morbidity and mortality [27]. Validation of Churchill’s performance using the GIAB Consortium reference sample [13] demonstrated that Churchill had the highest overall sensitivity (99.7%) and accuracy (99.9988%) of the pipelines assessed (Figure 3). In addition to speed and genotyping accuracy, Churchill’s deterministic performance sets a NGS analysis standard of 100% reproducibility without sacrificing data quality.

### Churchill enables population-scale genomic analysis in the cloud

Churchill not only optimizes the workflow for clinical analysis of single whole genome or targeted capture samples, but also for much larger research data sets. To demonstrate this, Churchill was used to analyze the 1KG raw dataset of 1,088 individuals [28] using the cloud (AWS EC2). Churchill was able to efficiently parallelize the entire analysis process, from FASTQ raw input data through multi-sample variant calling, generating population allele frequencies in under a week (Figure 5). A smaller scale simultaneous analysis of 61 human genomes was recently performed in 2 days with a Cray XE6 supercomputer, averaging 50 minutes per genome [29]. Through utilization of universally available on-demand cloud resources, Churchill completed analysis five times faster, averaging 9 minutes per genome, using one-third of the compute resources of the Cray supercomputer. Additionally, this undertaking demonstrates the feasibility of generating population allele frequencies specific to a given unified analysis approach, resulting in the discovery of approximately 3,000,000 novel indels. When utilizing Churchill, identification of rare pathogenic variation will be aided by supplementing 1KG consortium allele frequencies with Churchill-specific allele frequencies generated in this current analysis.

## Conclusions

Current approaches for data analysis of whole human genome sequencing data can take weeks to complete, resulting in bioinformatics overheads that exceed sequencing costs and represent a significant limitation. Churchill is a computational approach that overcomes these challenges, fully automating the analytical process required to take raw sequencing data through the complex and computationally intensive processes of alignment, post-alignment processing, local realignment, recalibration and variant discovery. A major contribution of this work has been the extensive study of strategies for parallelization of this workflow and implementation of a deterministic parallelization strategy that enables a load-balanced division of the entire analysis workflow. As a result, Churchill enables computationally efficient whole genome sequencing data analysis in less than 2 hours. In addition to rapid analysis of a single sample, Churchill optimizes utilization of available compute resources and scales in a near linear fashion, allowing population-scale genome analysis to be performed cost-effectively using cloud resources. As we look to the future, cloud computing will become indispensable for genomics [30-33]. Population-scale genomic studies are being made a possibility by declining sequencing costs and advances in sequencing technologies. Churchill eliminates the sequence analysis computational bottleneck and through use of cloud computing resources will make it possible to keep pace with the magnitude of genomic data that these new sequencers will create.

## Materials and methods

### Churchill parallelized workflow

Each of the required data processing steps was carefully examined (Figure S1 in Additional file 1) and optimal approaches for parallelized processing were determined. Alignment of individual reads to a reference genome is considered to be an embarrassingly parallel process as the 1 billion raw reads that are generated in sequencing a human genome can in theory be mapped to the reference genome independently of one another; the only constraint for paired-end reads is that both reads in a pair should be correctly oriented within proper distance. The remaining steps in the analysis workflow are not embarrassingly parallel by nature and, as such, required the development of novel approaches to achieve parallelization. One approach to enable a modest level of parallelization of the subsequent steps is to divide the analysis by individual chromosomes (22 autosomes (chromosomes 1 to 22) and two sex chromosomes (chromosomes X and Y)). However, doing so results in a significant load imbalance as the size of these chromosomes varies significantly, with chromosome 1 being approximately 5 times larger than chromosome 21 (Figure S3A in Additional file 1). In addition, limiting parallelization at chromosomal level restricts the use of processors to a total of 24, such that utilization of more than 24 CPU cores cannot improve performance.

To overcome this limitation of parallelization by chromosome, Churchill utilizes an approach that evenly subdivides the whole human genome into multiple regions with fixed boundaries (subregions), enabling a load balanced and independent execution of the local realignment, deduplication, recalibration and genotyping steps (Figure S3B in Additional file 1). However, there are four issues that arise with this strategy:

1. *Dependencies*. There are several points at which the results of processes run on individual segments of the genome are not independent. First, duplicate read removal requires the entire set of reads in sorted order so that any number of read pairs that have identical mappings can be reduced to a single pair. If one were to separate the data, read pairs must be kept together. A second point at which different segments depend on each other is during base quality score recalibration. Best practices suggest that a true baseline of base qualities requires examination of covariates across the entire sample.
2. *Parallelization*. Assuming these dependencies have been addressed, the issue then becomes how to parallelize these independent processes. One drawback of the computational techniques in genome resequencing and variant calling is the large memory requirements. Therefore, there may not be enough memory available to process as many segments as cores are available on the server. Also, load balancing is a concern.
3. *Determinism*. Ideally, introduction of a parallelization strategy should not produce different results depending on how the parallelization was implemented. If determinism is not maintained, then different results could occur based on the available resources at the time of analysis, creating an unacceptable situation for clinical applications where reproducibility and determinism are essential.
4. *Interchromosomal reads*. Most read pair distances will be normally distributed around a given insert size, which can vary between sequencing runs. Inherently, there will be outliers. These outliers could be either sequencing artifacts or improper mappings. In many cases, however, reads pairs with large insert sizes and those with each read of the pair on different chromosomes could indicate a structural variant and it is vitally important they are not disregarded. Shortcuts taken on the above described dependencies could result in lost information regarding interchromosomal reads.

In theory, the extremely large size of the human genome (approximately 3 billion base pairs) enables achievement of near-embarrassingly parallel execution of these steps. For example, dividing the genome into 3,000,000 base pair chromosomal subregions would enable execution of these steps in 1,000 parallel processes. The number of subregions created by Churchill can be specified by the user, although increasing this variable to twice the number of cores available for processing leads to improved load balancing. In order to ensure proper processing of regional boundaries, at both ends of each region, we include a 3 kilobase overlap of the adjacent region. This overlap acts as a ‘buffer zone’ to ensure appropriate detection of variants near or spanning region boundaries, as is possible in the case of indels. The resulting region and overlap boundary information is saved in the GATK intervals file format.

However, the post-alignment steps of the analysis process (local realignment, duplicate read removal, base quality score recalibration, genotyping and variant quality score recalibration) could not simply be performed on these subregions without significant refinement of each step to achieve high levels of parallelization without sacrificing data integrity and quality. The five steps of the Churchill workflow and the optimization that was performed are detailed below.

#### Step 1: parallelized alignment to a reference sequence

For the initial alignment step, BWA is utilized to perform reference genome alignment with the reads contained in paired FASTQ files. The speed of the process can be increased through utilization of inbuilt multithreading capabilities of the alignment algorithm by executing the aligner in multithreading mode (for example, using the *bwa aln -t* option to specify the number of threads). However, implementation of alignment within the Churchill pipeline utilizes an approach whereby the total raw input sequencing data (typically 400 to 800 million paired reads) is split into multiple smaller FASTQ files and aligned using multiple single-threaded parallel instances of the alignment algorithm. The number of paired-end FASTQ files generated during the sequencing run is controlled by the *--fastq-cluster-count* parameter of Illumina’s BCL-conversion process (CASAVA 1.8.2), which specifies the maximum number of reads per output FASTQ file. The default value of 4,000,000 works well with Churchill. However, decreasing the number of reads per FASTQ to 1,000,000 results in increased alignment speed due to better load balancing.

#### Step 2: parallelized generation and deduplication of subregional BAMs

At the heart of the Churchill pipeline is the novel algorithm we developed to convert the raw BAM files produced during alignment into subregions, enabling the parallel implementation of all of the subsequent analysis steps (Figure S4 in Additional file 1). This approach consists of 5 sequential steps:

1. *Split raw BAM by region.* The genome is split into *M* chromosomal subregions, where the value of *M* is defined by the desired level of parallelization. Utilization of the Churchill parallelized alignment approach generates *N* raw BAM files (derived from alignment of *N* pairs of input FASTQ files to the entire genome). These BAM files are split according to the coordinates of the subregions, yielding *M* × *N* split BAM files. Read pairs in which mates map to different subregions (including both interchromosomal and intrachromosomal reads) are temporarily transferred to separate split BAM files, one for each of the *N* input BAM files, identified as chrI.bam (‘I’ is short for inter/intrachromosomal mapping).
2. *Merge split BAMs by subregion.* For each of the genomic subregions, the *N* split BAM files corresponding to a given subregion are merged into *M* subregional BAM files, each containing all of the read pairs mapped within the boundaries of that subregion.
3. *Merge split chrI BAMs*. The *N* chrI BAM files are merged into a single genome-wide interchromosomal BAM file.
4. *Parallelized deduplication.* Duplicate reads are identified and removed from region and interchromosomal BAM files. Reads containing amplification errors may be represented in artificially high numbers and, as such, failure to remove these reads from the data set would have a significant negative effect on the final result by introducing variants that reflect these errors rather than true biological polymorphisms. The deduplication process identifies read pairs with identical external coordinates and subsequently reduces the data set to include only one copy of the duplicate sequence with highest mapping quality. Picard Tools MarkDuplicates is the tool most commonly utilized to identify duplicate reads both within and between chromosomes. Current best practices require that the deduplication process be performed using a single BAM file, containing all of the reads from the sequencing run. This is the approach utilized by the GATK-Queue analysis pipeline. However, in addition to this prolonged serial deduplication, the process of merging the BAM files into a single file cannot be parallelized. These processes result in lengthy single-threaded computations that substantially increase analysis run time. The Churchill algorithm overcomes this significant limitation by keeping interchromosomal reads together initially and deduplicating them. This step happens before the individual reads in the pair are merged by coordinates into the appropriate subregional BAMs. This innovative approach ensures proper deduplication of these interchromosomal reads and enables safe parallelization of the remainder of the deduplication process across both chromosomes and chromosomal subregions. In this way it is possible to achieve high levels of parallelization of the duplicate marking and removal process without compromising data integrity. The Churchill deduplicated BAM is indistinguishable from the results obtained from the lengthy process of post-alignment processing of a single merged genome-wide BAM file.
5. *Merge chrI reads with subregional BAMs.* The deduplicated interchromosomal paired reads are split according to subregion, and the individual reads are merged back into the appropriate subregion BAM according to the read coordinates. The resulting alignment files contain both appropriately deduplicated interchromosomal and regular reads. In addition, a copy of this chrI.bam file is kept as it may aid in detection and analysis of structural variants.

The final output of this step is multiple BAM files, one for every genomic subregion, which include appropriately mapped and deduplicated reads, thereby enabling parallelization of the subsequent steps.

#### Step 3: parallelized local realignment around indels

In the second post-alignment processing step, local read realignment is performed to correct for potential alignment errors around indels. Mapping of reads around the edges of indels often results in misaligned bases creating false positive SNP calls. Local realignment uses these mismatching bases to determine if a site should be realigned, and applies a computationally intensive Smith-Waterman algorithm to determine the most consistent placement of the reads with respect to the indel and remove misalignment artifacts [34]. The major advantage of Churchill in parallelizing local realignment is that all reads from a given sample are used to perform the local realignment, ensuring the greatest possible accuracy and improving novel indel detection. Moreover, applying sample-level local realignment across Churchill’s subregions results in significant improvements in speed.

#### Step 4: parallelization of base quality score recalibration

Each base of each read has an associated quality score, corresponding to the probability of a sequencing error. The reported quality scores are known to be inaccurate and as such must be recalibrated prior to genotyping, where they are used in the Bayesian genotype likelihood model employed by GATK’s UnifiedGenotyper [5]. After recalibration, the recalibrated quality scores in the output BAM will more closely correspond to the probability of a sequencing error. Moreover, the recalibration tool corrects for variation in quality with respect to machine cycle and sequence context, thus producing both more accurate and widely dispersed quality scores. Churchill uses GATK’s base quality score recalibration (BQSR) algorithm, which analyzes covariation among several features of a base, including the reported quality score, the position within the read and the preceding and current nucleotide (sequencing chemistry effect). These covariates are then applied through a piecewise tabular correction to recalibrate the quality scores of all reads in a given BAM file. However, according to the Broad’s best practices, BQSR requires a pool of all covariates from across the genome for proper calculation. Therefore, to ensure integrity of the recalibration process, Churchill merges the covariate results for each subregion so that each parallel recalibration instance has input data from the entire genome rather than just its region. The benefit of this approach is that it enables Churchill to use the entire dataset for recalibration purposes, improving accuracy and avoiding downsampling, which will lead to non-determinism.

#### Step 5: parallelization of variant calling

In the penultimate step of the Churchill analysis process, variant calls can be generated with GATK UnifiedGenotyper [5], GATK HaplotypeCaller [15] or Freebayes [23] using the analysis ready reads generated during recalibration. Churchill is capable of implementing these algorithms on both single sample data and multi-sample data, where variant information from all samples in a given experiment is utilized to improve genotyping accuracy. Due to the overlapping buffer zones at the ends of each region, it is possible that a variant occurring in one of these zones may be called twice: once in the region to which it belongs and once in the overlap zone of the adjacent region (Figure S5 in Additional file 1). This is corrected by assigning the variant to the appropriate subregion and removing its buffer-zone duplicate from the final merged raw variants file. This determination is made based solely on the location of the variant call and its position relative to the fixed subregion boundaries. The raw genotype calls from each subregion are concatenated into genome-wide VCF files for both SNPs and indels ready for down-stream analysis and interpretation.

### Churchill implementation

A schematic representation of the entire Churchill process is shown in Figure S2 in Additional file 1. Compared with alternative analysis pipelines, implementation of Churchill is simpler, faster, and more widely applicable to various shared memory/distributed high performance computing (HPC) clusters. Churchill only requires a small number of pre-installed components. Python (with PySam), BWA, Samtools, Picard Tools, and GATK are the only required software not included in Churchill. Setup and execution are performed with a single command. Churchill is implemented as a mixture of Bash and Python scripts, linking and preparing for parallelization the inputs and outputs of BWA, Picard, SAMTools, and GATK. A single configuration file defines the paths to raw data, installed software, required database files, and delivery directories. Churchill is initialized and executed using a single python script, ‘churchill.py’, and a Cython-compiled C library. Churchill begins by creating the scripts required to run the entire pipeline and then proceeds to execute (or submit to the job scheduler) the scripts in the desired parallelization method (shared memory, GNU make, Sun Grid Engine (SGE), or Portable Batch System (PBS)) specified by the user in an argument to ‘churchill.py’. To ensure that Churchill would be of utility to the widest number of researchers, the pipeline was developed such that it could be executed with three of the most commonly utilized environments for distributed computing: shared memory machine or server with explicit task creation, shared memory machine or server with task creation by GNU Make, and HPC clusters and cloud implementations that support distributed Make, such as PBS and SGE (Table S1 in Additional file 1). As such, Churchill is compatible with a wide range of Linux systems, including high-performance workstations, small single servers, moderate in-house clusters with shared or non-shared memory servers, large HPC systems housed at supercomputing centers (including the Ohio Supercomputing Center and the Pittsburgh Supercomputing Center) and in the cloud. The software is available for download at [35].

### Data availability

Sequence data were generated in the Biomedical Genomics Core, The Research Institute at The Nationwide Children’s Hospital. Initial development and testing of Churchill was conducted using Illumina HiSeq 2000 100 bp paired-end whole genome sequencing data sets with 30× average coverage. Informed consent was obtained from study subjects or parents of subjects less than 18 years of age (assent was obtained from subjects 9 to 17 years of age) under protocols approved by the Institutional Review Board (IRB) at Nationwide Children’s Hospital (protocol number IRB11-00215). Analysis timing metrics and validation were performed using NA12878 sequence data deposited in the Sequence Read Archive (accession ERP001229) down-sampled to 30× coverage as described below. The phase 1 1KG data are available through the consortiums FTP sites (see [36] for details). The NIST benchmark SNP and indel genotype calls generated by the GIAB Consortium can be downloaded from [37]. All experiments have been conducted according to the principles expressed in the Declaration of Helsinki.

### Validation

Validation was performed using FASTQ files from the Sequence Read Archive study ERP001229 for whole human genome sequencing of the 1KG CEU female NA12878 (Illumina HiSeq 2000 paired-end 100 bp reads, split into 431 pairs of FASTQ files, each containing 2,000,000 reads). The VCF files produced from these data by Churchill, GATK-Queue and HugeSeq were compared with NIST benchmark SNP and indel genotype calls generated by the GIAB Consortium [13]. First, VCFs were filtered to remove low quality variant calls as indicated by a ‘LowQual’ flag generated by the given pipeline. Second, the VCF was filtered to the GIAB callable regions using the vcflib [38] tool vcfintersect with the BED file provided by GIAB. Third, complex variants were decomposed into a canonical SNP and indel representation using the vcflib tool vcfallelicprimitives. Finally, VCF files were converted to tables and compared with the GIAB validation dataset (version 2.18) using custom scripts in R.

### Profiling and benchmarking

In addition to measuring the running time, we recorded the CPU utilization profile using the collectl utility [39], a comprehensive tool to measure the performance of a linux system. CPU, memory, disk, and network usage were measured at 10 s intervals. The output was then parsed and plotted using scripts customized for this purpose.

### Analysis with the bcbio-nextgen pipeline

The bcbio-nextgen run was performed using version 0.7.9 of the software that was installed using the provided installer script (bcbio_nextgen_install.py). After installation, the GATK software was upgraded using the provided upgrade script (bcbio_nextgen.py upgrade) to version 3.2-2 so that GATK’s HaplotypeCaller could be used. The run was performed on a single r3.8xlarge AWS EC2 instance. The run requested 32 cores to be used (-n 32) since 32 cores were available on the r3.8xlarge instance. This resulted in BWA-MEM being assigned 16 cores (-t 16) and sambamba being assigned 16 cores (-t 16).

### Churchill processing of 1000 Genomes Project data

To process each sample, the input FASTQ files for the sample were first copied from the 1000genomes S3 bucket to local storage on an EC2 instance. These input files were then processed by the Churchill pipeline to produce a set of realigned and recalibrated BAM files, one for each Churchill region. Finally GATK’s UnifiedGenotyper was run over the realigned and recalibrated BAM files from each sample to produce a single multi-sample VCF. A hard filtering strategy was employed similar to that used by the 1KG group’s original analysis of these data [28]. The single multi-sample VCF was filtered to remove indels with an unfiltered depth over all samples (DP) <2,566, DP >16,320, inbreeding coefficient < -0.8, quality by depth (QD) <1.5, or strand bias estimated using Fisher’s Exact Test (FS) >200. SNPs were removed with DP <2,566, DP > 16,320, QD <1.5, root mean square of the mapping quality of reads across all samples (MQ) <30, FS >80, or consistency of the site with strictly two segregating haplotypes (HaplotypeScore) >13. The VCF file is available to download at [35].

## Additional file

Additional file 1:
**This file contains five supplementary figures further detailing the Churchill balanced parallelization method and one supplementary table comparing parallelization environments and methods.**

## Abbreviations

1KG: 1000 Genomes Project
AWS: Amazon Web Services
BAM: binary sequence alignment map format
BQSR: base quality score recalibration
BWA: Burrows-Wheeler Aligner
CPU: central processing unit
.EC2: Elastic Compute Cloud
GATK: Genome Analysis Toolkit
GIAB: Genome in a Bottle Consortium
HPC: high performance computing
indel: small deletion and insertion
I/O: input/output
NGS: next generation sequencing
NIST: National Institute of Standards and Technology
PBS: Portable Batch System
SAM: sequence alignment map format
SGE: Sun Grid Engine
SNP: single nucleotide polymorphism
VCF: variant call format
