# Supplementary material for: Churchill: an ultra-fast, deterministic, highly scalable and balanced parallelization strategy for the discovery of human genetic variation in clinical and population-scale genomics
Source: Genome Biol. 2015 Jan 20;16(1):6. doi: 10.1186/s13059-014-0577-x (PMC4333267; doi:10.1186/s13059-014-0577-x)
Supplement: Additional file 1: — This file contains five supplementary figures further detailing the Churchill balanced parallelization method and one supplementary table comparing parallelization environments and methods. [file 13059_2014_577_MOESM1_ESM.pdf]

-- ADDITIONAL ONLINE FILE 1 --

Kelly *et al.* Genome Biology 2015, 16:6. doi: 10.1186/s13059-014-0577-x

SUPPLEMENTARY FIGURES

Churchill: An Ultra-Fast, Deterministic, Highly Scalable and Balanced Parallelization Strategy for the Discovery of Human Genetic Variation in Clinical and Population-Scale Genomics

Benjamin J Kelly[1], James R Fitch[1], Yangqiu Hu[1], Donald J Corsmeier[1], Huachun Zhong[1], Amy N Wetzel[1], Russell D Nordquist[1], David L Newsom[1] and Peter White[1,2*]

[*] Correspondence: peter.white@nationwidechildrens.org

[1] Center for Microbial Pathogenesis, The Research Institute at Nationwide Children's Hospital, 700 Children's Drive, Columbus, OH 43205, USA

[2] Department of Pediatrics, College of Medicine, The Ohio State University, Columbus, Ohio, USA

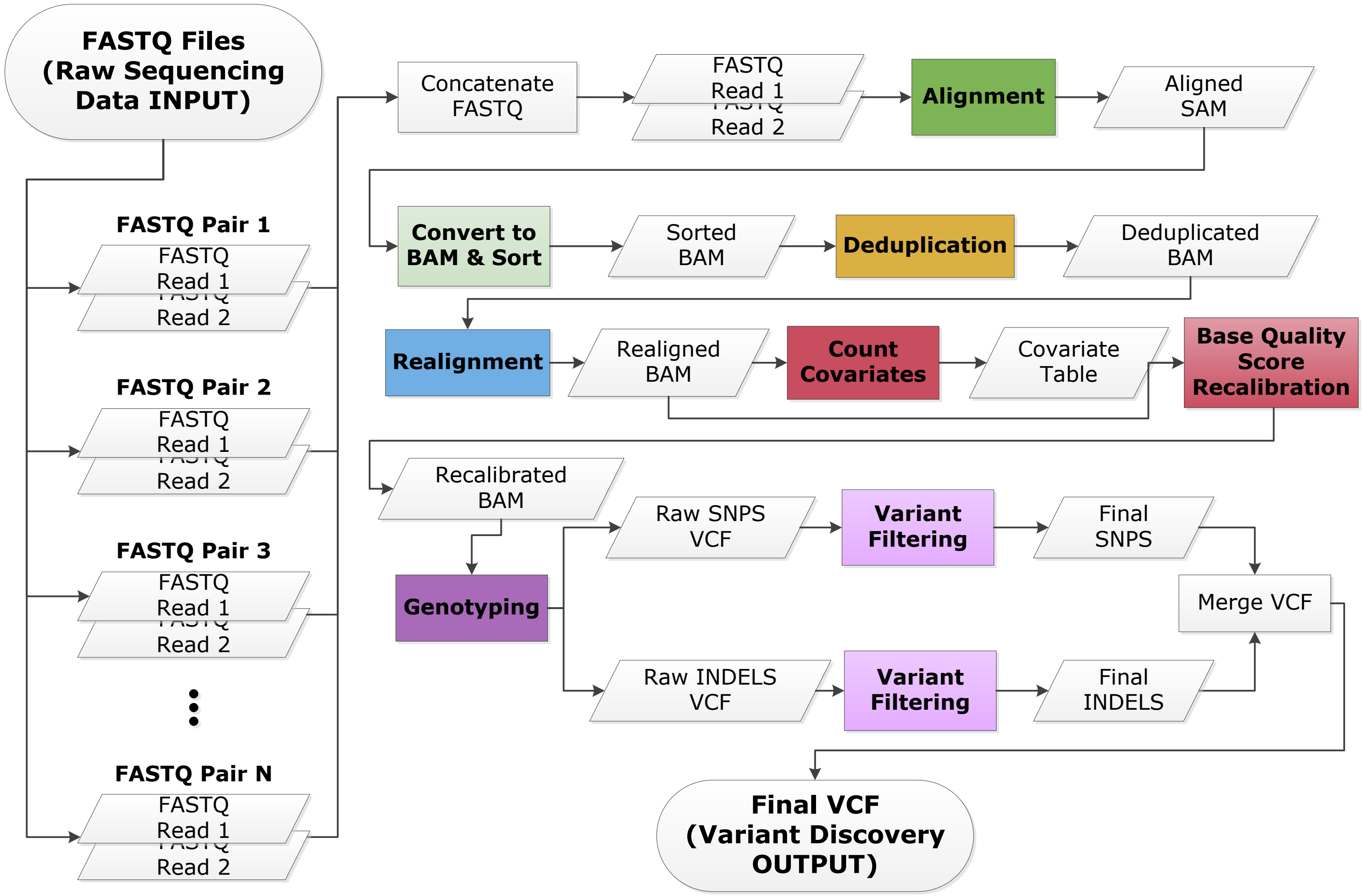

Supplementary Figure 1. Serial data processing steps for the analysis of human genome resequencing data. Current best practices for making SNP and indel calls requires five sequential steps: (1) initial read alignment; (2) removal of duplicate reads; (3) local realignment around indels; (4) recalibration of base quality scores; and (5) variant discovery and genotyping. These steps are the same for deep and low-pass whole genomes, whole exomes and targeted resequencing. Color shading is included to allow cross referencing of each of these steps to the detailed Churchill workflow presented in **Supplementary Figure 2.**

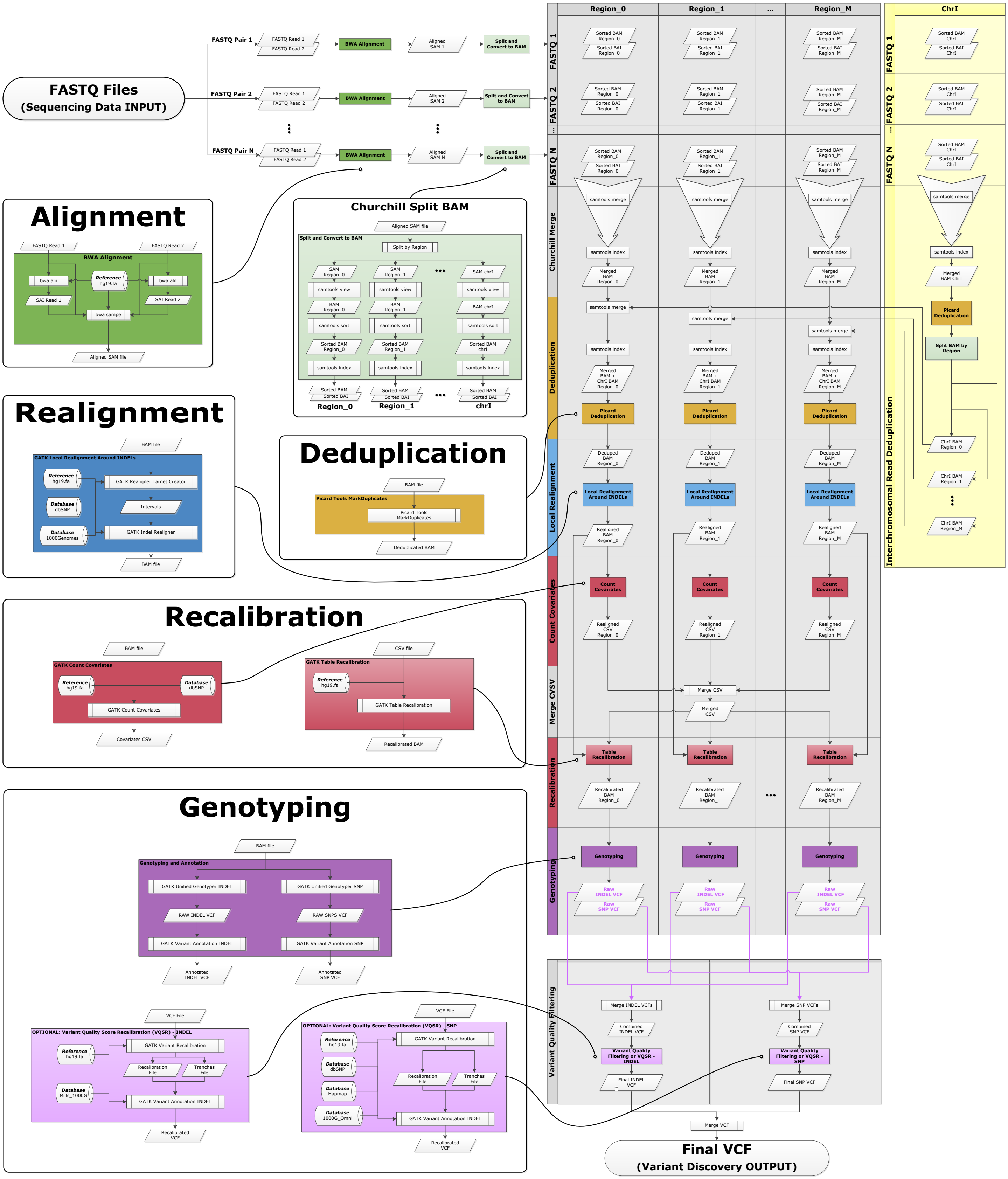

Supplementary Figure 2. Full schematic overview of the Churchill pipeline. High resolution interactive graphic designed to only be read online – use Acrobat zoom to view details.

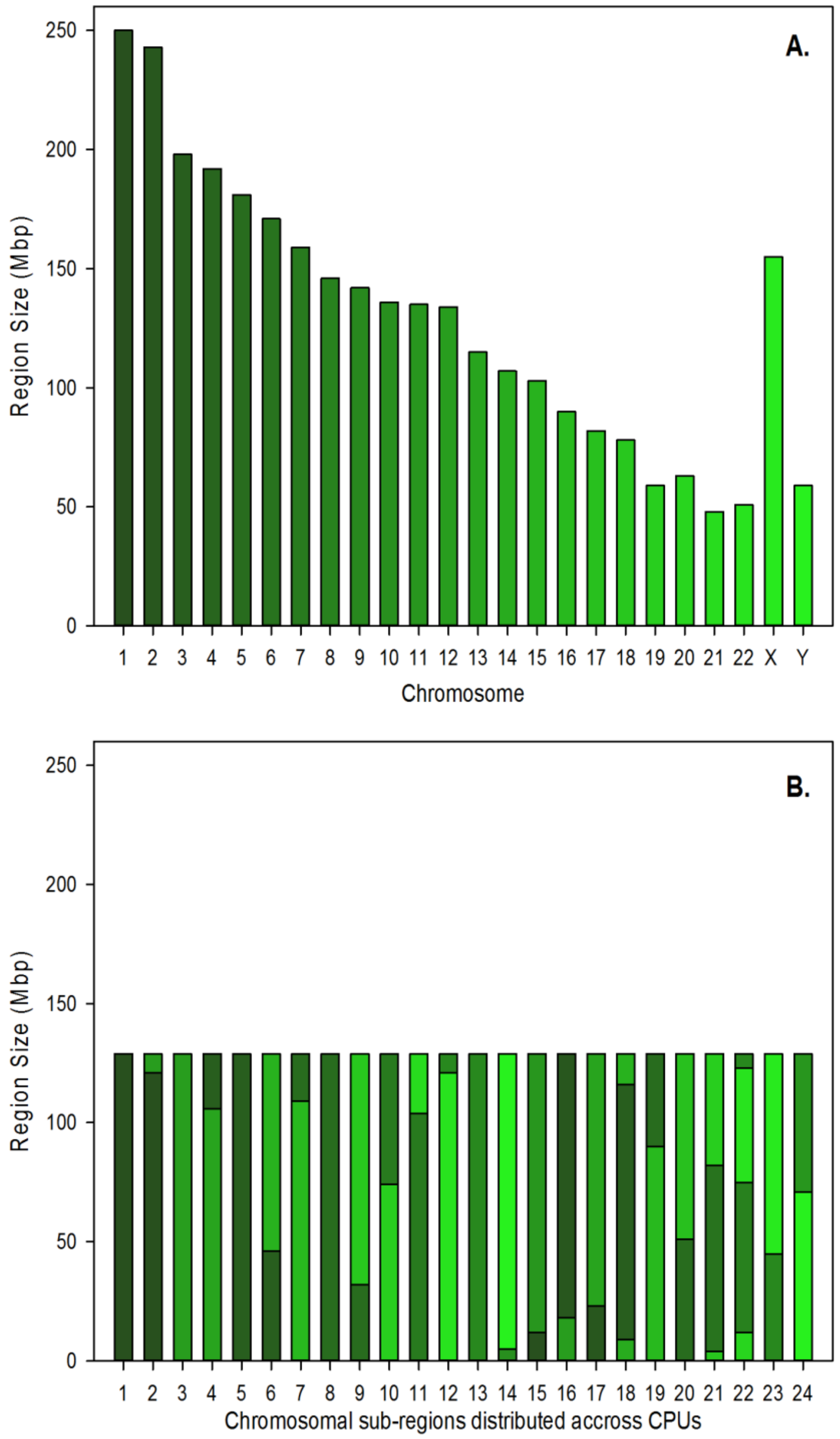

Supplementary Figure 3. Churchill splits the genome into chromosomal subregions, thereby equilibrating load balancing and enabling high levels of parallelization. Parallelization by chromosome suffers from inherent load imbalance, due to the varying sizes of the human chromosomes (A). However, utilization of chromosomal subregions enables equilibration of the analysis load across all available processors (B).

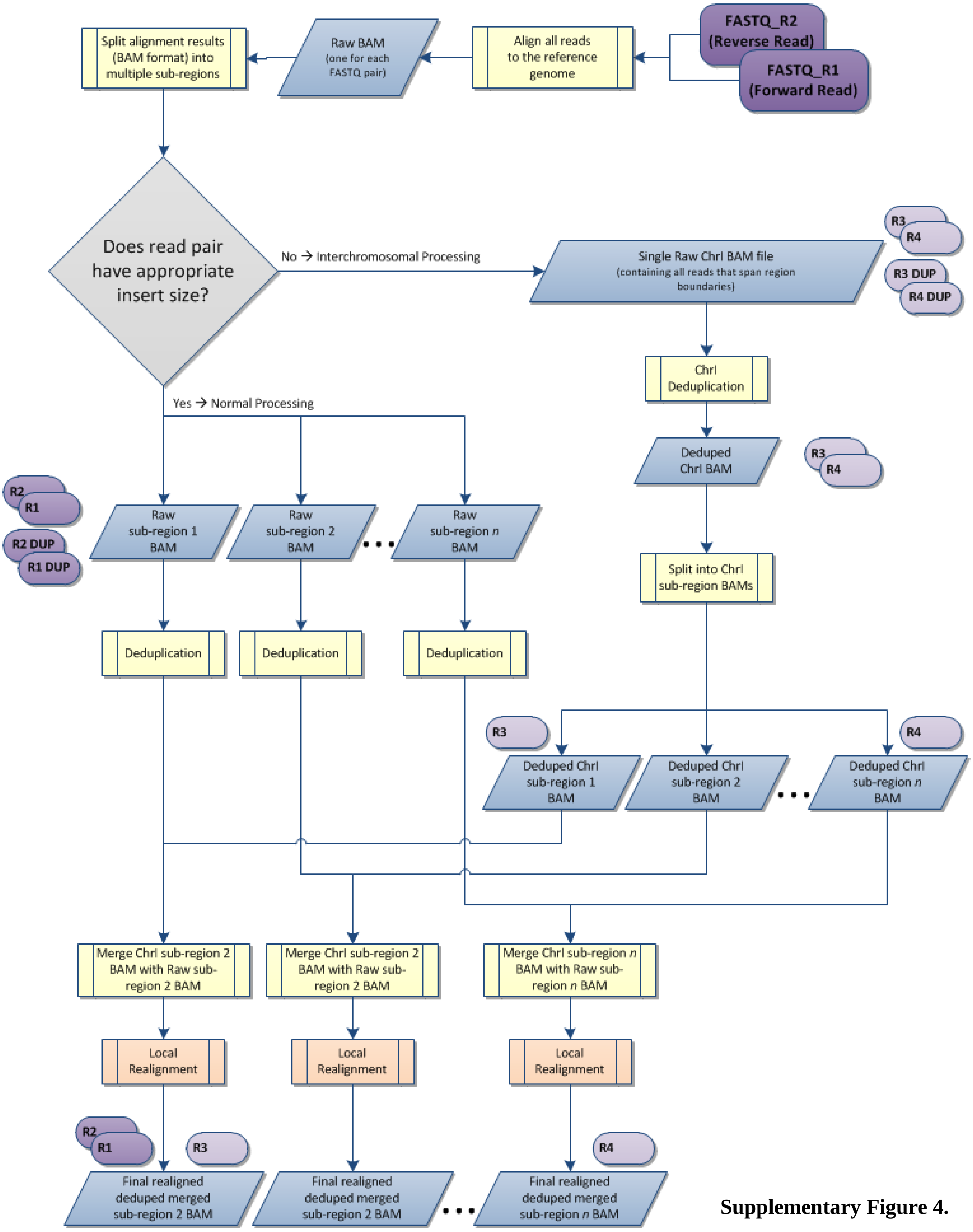

Supplementary Figure 4.

Supplementary Figure 4. Churchill parallelized deduplication algorithm. Following alignment, reads are split into multiple subregion BAM files. If both reads in the pair map to the same region they are placed into the appropriate subregion BAM file. Otherwise, the reads are placed in the interchromosomal (ChrI) BAM file. Once the raw aligned reads have been processed, the interchromosomal reads can then be correctly deduplicated. The deduplicated interchromosomal reads are individually merged back into their appropriate subregion BAM. These merged subregion BAMs then undergo local realignment and deduplication, creating deduplicated subregion BAMs ready for the recalibration and genotyping steps.

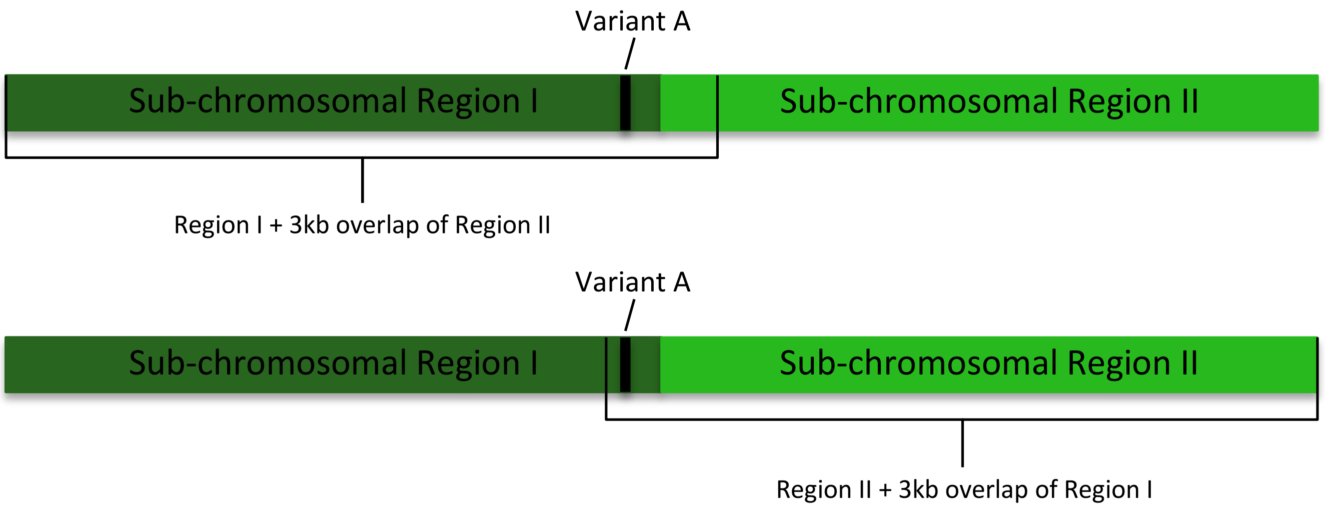

Supplementary Figure 5. Illustration of Churchill subregion processing. Chromosomes are split into subregions for the processes of realignment, duplicate removal, base quality score recalibration, and genotyping. If duplicate variant calls are made in the overlapping regions, Churchill assigns the variant to the correct subregion. These buffer zones allow data integrity to be maintained while enabling parallelization of the data processing steps. For example, Variant A called in the last 3 kilobases of Region I is called twice, once in the processing of Region I, once in the processing of Region II; it is assigned to Region I.

	Parallelization environment		
**Parallelization method**	**Shared memory**	**PBS**	**SGE**
Xargs	Yes	No	No
GNU Make	Yes	Yes (via distmake)	Yes (via qmake/distmake)
Qsub	No	Yes	Yes

Supplementary Table 1. Comparison of parallelization environments vs. parallelization methods.
